# Neurological symphony: post-acute COVID-19 syndrome, an innovative pathophysiological exploration from neuraltherapeutic medicine

**DOI:** 10.3389/fnint.2024.1417856

**Published:** 2024-07-12

**Authors:** Carlos Bustamante, Laura Bibiana Pinilla Bonilla, Juan Carlos Restrepo

**Affiliations:** ^1^Institute for Advanced Integrative Medicine, Medellin, Colombia; ^2^Faculty of Medicine, National University of Colombia, Bogotá, Colombia; ^3^Research and Innovation Center, Integrated Subnetwork of Health Services of South, Bogotá, Colombia; ^4^University of Santiago de Cali, Cali, Colombia

**Keywords:** neural therapy, local anesthesia, inflammation, neuromodulation, post-acute COVID-19 syndrome

## Abstract

The SARS-CoV-2 pandemic has affected 771 million people and caused 6.9 million confirmed deaths as of November 2023. Beyond the adversity, a crucial and less-explored chapter unfolds: adaptive sequelae. These have altered social, mental, and emotional conditions, leaving an imprint on biological systems. While some cases fully resolve the pathological process post-acute infection, others persist with symptoms, posing a challenge that underscores the need to comprehend pathophysiology from innovative perspectives. The article delves into “Long COVID” or Post-Acute COVID-19 Syndrome (PACS), where symptoms persist for ≥4 weeks irrespective of initial severity. Risk factors include a history of severe illness, in-hospital management, and intensive care. This article also explores theories, derived from various experimental models, that have demonstrated the involvement of the nervous system in coordination with the psychoneuroimmunoendocrine axes in the expression of inflammation. It is posited that PACS involves processes of peripheral and central sensitization (corticalization), facilitating dishomeostasis and the chronicity of the inflammatory process. In this context, various therapeutic strategies grounded in modulating the inflammatory reflex are reviewed, primarily through the infiltration of local anesthetics via linear and non-linear approaches. Neural therapeutic use is considered to stimulate the regulatory inflammatory circuits coordinated by the neuroimmune-endocrine system.

## Introduction

The SARS-CoV-2 pandemic (Ritchie et al., 2022) has affected 771 million people and caused 6.9 million confirmed deaths as of November 2023 (WHO, 2022). Beyond the adversity, a crucial and less-explored chapter emerges: its adaptive aftermath. These have altered social, mental, and emotional conditions, leaving an imprint on biological systems. Yet, the fact that some cases completely resolve the pathological process while others persist with symptoms after acute infection remains an enigma. This phenomenon poses a challenge that underscores the need to comprehend the pathophysiology from innovative perspectives, providing tools to address the post-pandemic landscape.

Persistent symptoms following acute infection, occurring between 4 to 12 weeks, are termed Long COVID or post-acute COVID sequelae. When these symptoms persist for ≥4 weeks, it is referred to as Post-Acute COVID-19 Syndrome (PACS) (Nalbandian et al., 2021; Shah et al., 2021; Greenhalgh et al., 2022; Li et al., 2023). These symptoms do not directly correlate with the severity of the acute infection, but there are risk factors such as a history of severe illness from SARS-CoV-2, in-hospital management, and intensive care. It is noteworthy that PACS can manifest in individuals with asymptomatic disease or without prior confirmed infection (Nalbandian et al., 2021). Despite the high effectiveness of vaccination in preventing severe acute illness from SARS-CoV-2, some authors have reported an increased risk of PACS if infection occurs within 14 days post-vaccination (Al-Aly et al., 2022) and on the other hand (Saheb Sharif-Askari et al., 2024) in a cohort study in 28,375 non-hospitalized adult patients diagnosed with mild to moderate COVID 19 in Dubai, emphasize the potential benefits of pre-COVID vaccination and timely treatment in the prevention of Long COVID.

To date, there is no diagnostic gold standard for Post-Acute COVID-19 Syndrome (PACS), and its symptoms are highly varied; moreover, they may be associated with other health issues (Nalbandian et al., 2021), complicating the diagnostic process. The multiorgan sequelae of PACS exhibit a broad spectrum of clinical manifestations (see Table 1), with the most common being fatigue (80%), weakness after physical exertion (73.3%), and cognitive impairment (58.4%), among others (Davis et al., 2021). Additionally, the pathophysiological mechanisms and effective therapeutic options have yet to be clearly defined (Nalbandian et al., 2021).

**Table 1:** Persistent COVID-19 Symptoms, indicating damage caused by specific and non-specific mechanisms following SARS-CoV-2 infection.

**General** Fatigue Post-exertional discomfort Fever Deterioration of quality of life
**Cardio-respiratory** Shortness of breath or trouble breathing Cough Chest pain Palpitations Postural orthostatic tachycardia syndrome
**Neurological** Myalgia encephalomyelitis and chronic fatigue syndrome Difficulty thinking or concentrating Headache Vision changes Sleep disorders Orthostatic hypotension “Pins and needles” paresthesias Smell or taste disorder Depression or anxiety
**Kidney** Decreased glomerular filtration rate
**Gastrointestinal** Diarrhea Constipation Stomachache Heartburn Elevation of aminotransferases Pancreatitis Exocrine and endocrine pancreatic dysfunction
**Osteo-articular** Myalgias-arthralgias Muscular weakness Sarcopenia
**Blood vessels** Thrombogenesis
**Dermatological** Rash Alopecia Urticaria
**Reproductive system** Changes in menstrual cycles Decreased libido Difficulty ejaculating

Modified reference: NIH COVID-19 treatment guideline (NIH, April 21, 2021) (Nalbandian et al., 2021; Li et al., 2023).

In a systematic review, 73% of individuals exhibited at least one persistent symptom 6 months after SARS-CoV-2 infection (Nasserie et al., 2021). In an observational study involving 3,762 patients from 56 countries, 62% presented with at least one persistent symptom at the 6-month mark (Davis et al., 2021). Due to the variability in clinical presentations and the diagnostic complexity of Post-Acute COVID-19 Syndrome (PACS), the prevalence remains uncertain, ranging between 5 and 80% (National Institutes of Health, 2021). The heterogeneity across studies underscores the necessity for more standardized diagnostic designs and prolonged follow-up.

**Figure 1:**
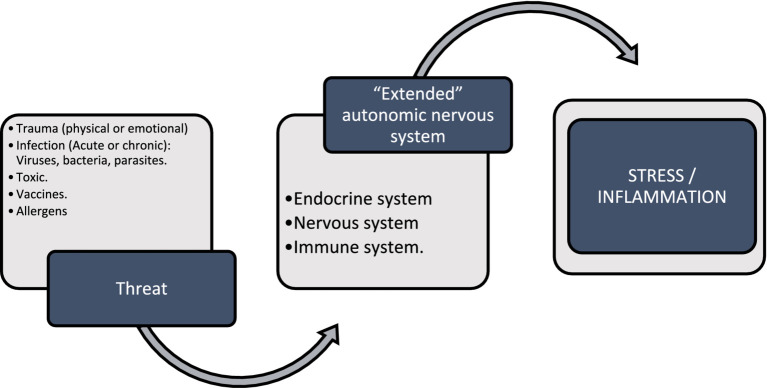
Nonspecific response to threatening stimuli such as trauma, microorganisms, toxins, vaccines, allergens that stimulate the extended autonomic nervous system or neuroimmunoendocrine system; and it responds as a unit through different cellular pathways and chemical communication with stress and inflammation.

This article reviews the pathophysiology of Post-Acute COVID-19 Syndrome (PACS), focusing on mechanisms of the nonspecific response to disease, particularly inflammation. It explores the nonspecific response to threats (see Figure 1) through Nervism theory, highlighting neurogenic dystrophy as a fundamental component of all diseases according to A.D. Speransky and an indispensable precursor for the development of pathologies according to G.N. Kryzhanovskysirve. Tracey’s inflammatory reflex, Chiu’s neurogenic inflammation, and Klein’s neuroimmune axis are analyzed, comparing them with the central and peripheral sensitization model. The integration of these processes into the psychoneuroimmunoendocrine system is described. Additionally, other specific responses to SARS-CoV-2 related to symptom persistence are briefly addressed. Finally, it emphasizes how Neuraltherapeutic Medicine, using local anesthetics (AL), can modulate the nonspecific response and neurogenic inflammation.

## Nervism: a physiological doctrine centered around the nervous system

Throughout 170 years of history, the so-called Russian nervism school has left an indelible mark on the study of the nervous system (NS). Initiated by the synthetic physiology of Pavlov Smith G. P. (2000) and developed by Speransky (Speransky, 1954), Orbeli (1938), and Bykov (Bykov et al., 1958), this school has solidified the differential approach. Its concept of the living system as a biologically indivisible functional unit underscores the NS as the supreme director of all physiological and pathological processes (Sechenova et al., 1993; Ariza Tarazona et al., 2020).

Speransky (1954) developed a theory aiming to generate a profound understanding, based on experimental foundations, of organisms’ responses to external irritating stimuli. This theory is grounded in reflex mechanisms of the nervous system, proposing a novel mechanism for the genesis of diseases overall. According to this theory, injurious stimuli have the potential to induce extreme irritation that affects tissue resilience—a phenomenon common to all pathophysiological processes known as the nonspecific response to damage, conceptualized by the theory of neurogenic dystrophies (Sechenova et al., 1993). This process, termed neurogenic tissue dystrophy, is caused by inadequate nervous influx to the involved tissue, disrupting the physiological cellular response and increasing tissue fragility, predisposing it to diseases (Akimov and Kositsyn, 2005).

On the other hand, Bykov and Anichkov suggested the occurrence of a reflex dystrophy associated with irritation of reflexogenic zones (Забродин, 1999), possibly linked to associated neuroanatomical circuits.

The influence of the nervous system (NS) on tissue homeostasis, along with its interaction with various physiological and pathophysiological phenomena, has been substantiated by other researchers. Its impact extends to cell division and differentiation (Bustamante et al., 2023), the modulation of hormonal or pharmacological responses (Sechenova et al., 1993; Akimov and Kositsyn, 2005), as well as changes in tissue ultrastructure and cytochemical profile (Tweedle et al., 1975; Kositsyn, 1978). Furthermore, regulatory effects on gene expression in tumors and their surrounding microenvironment have been observed (Cole et al., 2015). Its documented influence spans cellular metabolic processes (Pavlov and Tracey, 2012), thermogenesis, modulation of immunity, acute and chronic inflammation, and tissue repair (Tracey, 2002; Pavlov and Tracey, 2012; Klein Wolterink et al., 2022).

These findings support that the nervous system (NS) maintains the structural stability, functions, energy, and plastic processes of cells, tissues, organs, and the organism as a whole (Забродин, 1999).

Secondary reflex responses to irritative processes coordinated by the NS involve nonlinear physiological mechanisms that can be summarized into three main types (Speransky, 1954; Engel et al., 2022):

1. *Direct local irritation:* is generated by the direct irritation of the tissue and its corresponding nociceptors.
2. *Segmental metameric irritation:* is grounded in the embryogenesis of various tissues. This is primarily interconnected through the nervous system (NS), establishing segmental circuits that regulate function and communication between diverse structures. Reflex irritation from these circuits can trigger responses that impact innervation and, consequently, the function of related anatomical structures.
3. *Meta-segmental irritation:* is a reflex response that extends beyond the segment and lacks a local or embryogenic basis. It is currently referred to as “neuromodulatory trigger points” according to Engel et al. (Engel et al., 2022). Other authors have described it as a “neural interference field,” especially in the context of the Neural Therapy school according to Huneke (Dosch and Dosch, 2007). Although its pathophysiological mechanism is not fully understood, it pertains to cortical coupling phenomena described by temporal associations of the nervous system, such as Pavlov’s conditioned reflex (Bykov et al., 1958), as well as polysegmental neuroanatomical connections (Engel et al., 2022).

These connections can influence irritation and reflex responses in distant anatomical sites, emphasizing the complexity of interactions within the nervous system.

## Inflammatory reflex, neuroimmune circuit, and neurogenic inflammation

**Figure 2:**
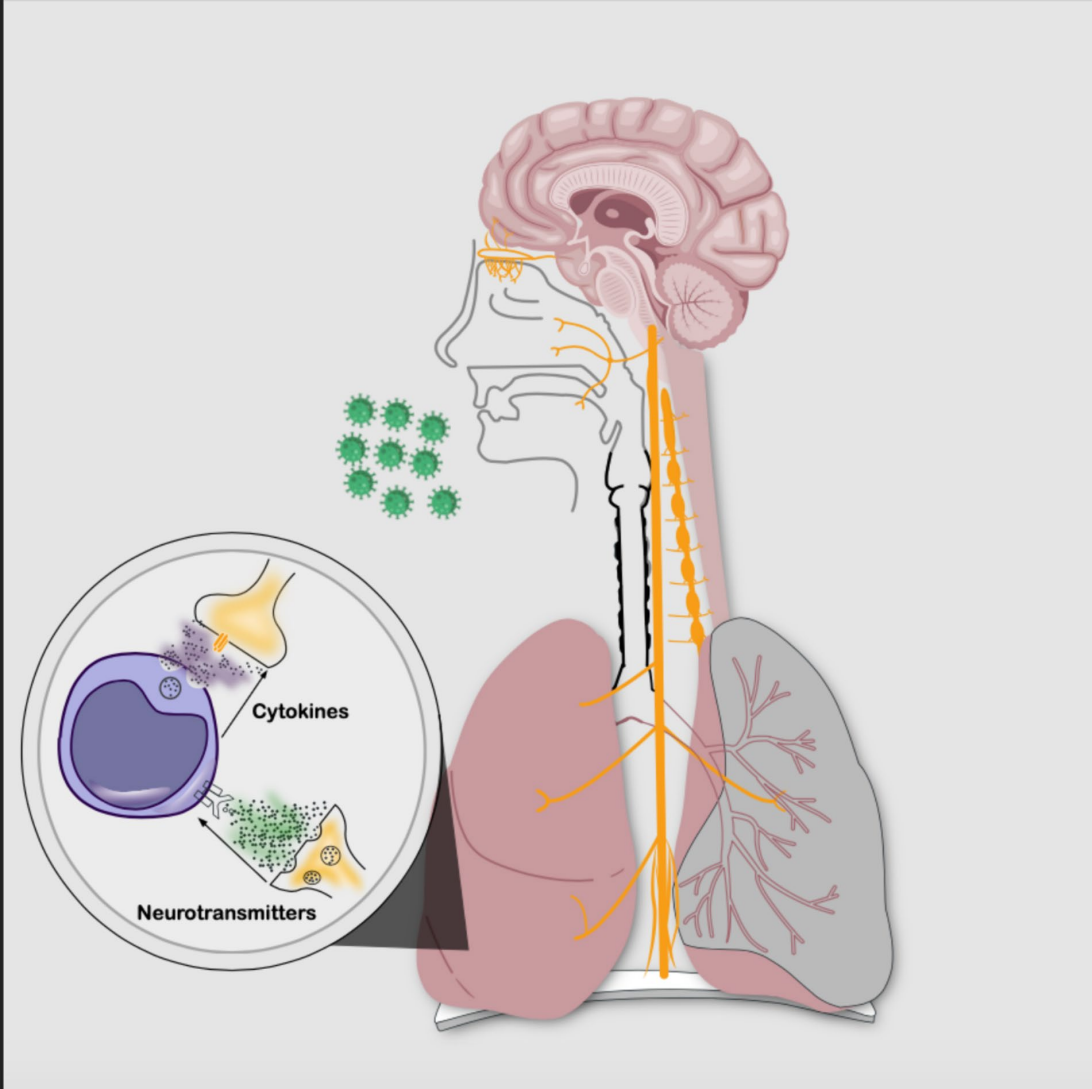
Neuroimmune circuit: bidirectional communication between the nervous system and the immune system through cellular phases and chemical signaling. The intricate interplay is established by neurotransmitters binding to immune cell membrane receptors and also by cytokines interacting with nerve cell membrane receptors. (Source: Own elaboration).

Bustamante et al. (Bustamante et al., 2023), Tracey (Tracey, 2002), Klein et al. (Klein Wolterink et al., 2022), and Chiu et al. (Chiu et al., 2012) have proposed that the nervous system (NS) and the immune system interact, forming the neuroimmune circuit to regulate homeostasis through the inflammatory reflex, also known as the neural phase of inflammation (see Figure 2). This process modulates the innate and adaptive immune response within seconds, acting as an autonomic reflex. The afferent pathway detects immune products secondary to tissue injury or infection through nerve terminals (Tracey, 2002), while the efferent pathway regulates the phenotypic expression of immune cells and the release of cytokines. The parasympathetic pathway is associated with anti-inflammation, whereas the sympathetic pathway is pro-inflammatory (Tracey, 2002; Chiu et al., 2012; Davidson et al., 2014; Cook et al., 2018; Klein Wolterink et al., 2022); this interaction becomes even more complex with a system of cells and chemical mediators such as neurotransmitters, neuropeptides, and cytokines interacting with each other (Goldstein, 2020).

Autonomic dysfunction of this inflammatory reflex, secondary to nociceptor sensitization, leaves the NS in a hyper-vigilant state (Cook et al., 2018), also known as “hyperinflammatory reflex” according to Engel et al. (Engel et al., 2022). This state triggers an axonal reflex that can induce neurogenic inflammation, leading to an increase in oxidative stress and its adverse effects. These alterations affect the biophysical properties of neuronal membrane potential, contributing to the development of intractable pain (Cook et al., 2018). In animal models, enteric inflammation affects sympathetic and sensory innervation, resulting in hypersensitivity associated with inflammatory bowel disease (Meade and Garvey, 2022).

## Phenomenon of central and peripheral sensitization and neurogenic inflammation

Peripheral sensitization is defined as an increase in the sensitivity of nociceptors at the peripheral nerve endings of the body, facilitating the onset of pain and other discomfort. This phenomenon leads to changes in membrane potential and electrochemical balance, resulting from alterations in the redox balance, primarily induced by an increased production of free radicals (Davidson et al., 2014). Meanwhile, central sensitization at the spinal and cerebral levels amplifies nociceptive signals from the nervous system. Both phenomena generate inflammatory responses that contribute to the injury and dysfunction of the affected tissues (Davidson et al., 2014).

## From the extended autonomic nervous system to psychoneuroimmunoendocrinology

The role of the nervous system (NS) in the trophic processes of all biological systems in the human body is closely tied to the information systems that integrate the complexity of organisms. Once considered isolated feedback systems, Goldstein (Goldstein, 2020) now refers to it as the “extended” autonomic nervous system with neuroimmuneendocrine circuits that interact harmoniously to maintain homeostatic balance. The cellular and chemical communication phase of this neuroimmuneendocrine system (NIES) shares common receptors and ligands, constituting interdependent axes of bioregulation (González-Díaz et al., 2017; Goldstein, 2020).

**Figure 3:**
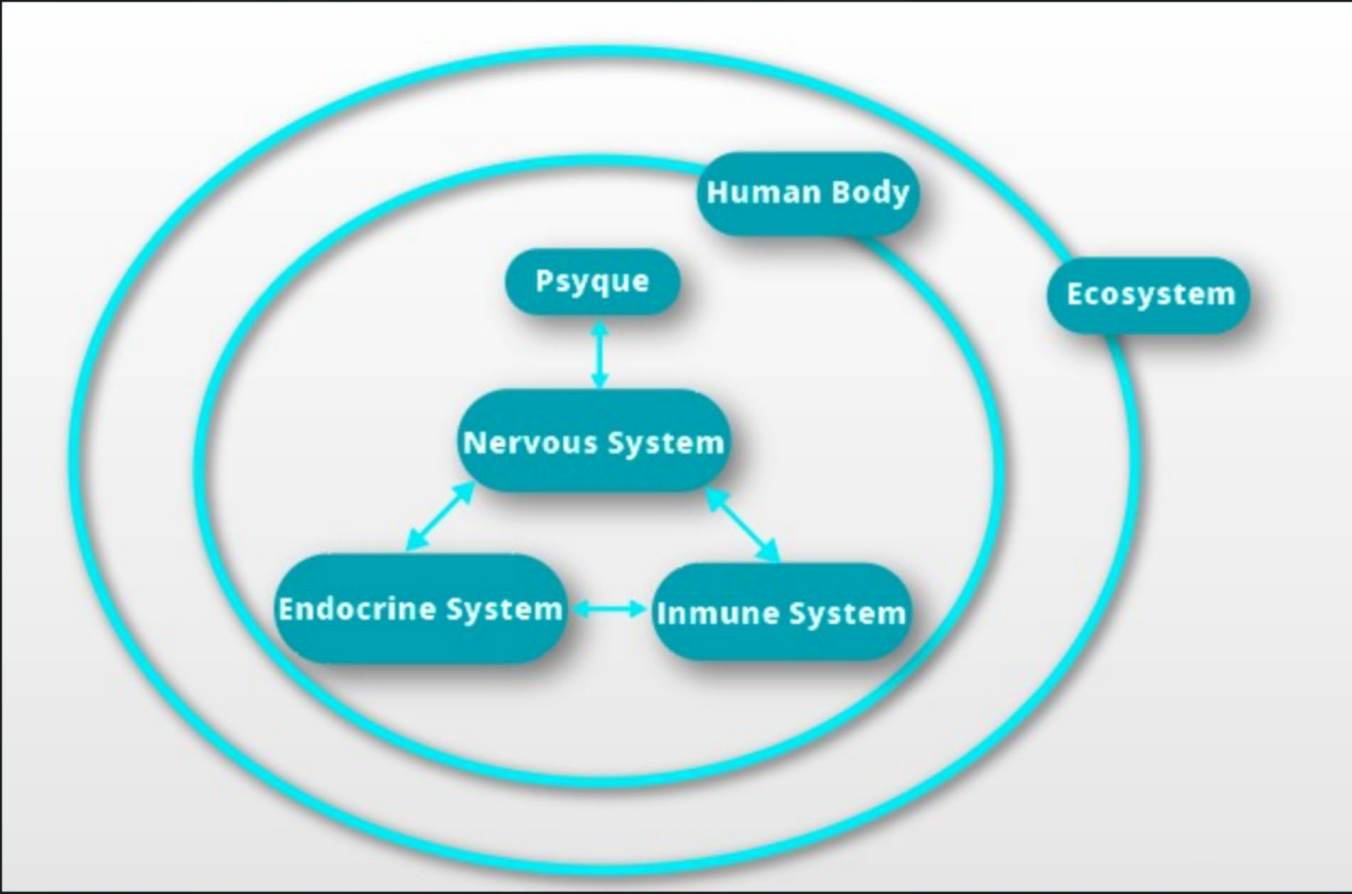
Information systems or psychoneuroimmunoendocrine axis. At the top within the human being, the psyche or consciousness harbors thoughts and emotions. The communication interface between the psyche and the physical body is carried out through the nervous system. The neuroimmune axis transmits chemical signals through cytokines and neurotransmitters. The neuroendocrine axis involves chemical transmitters, such as hormone-releasing factors and hormones. The immunoendocrine axis comprises chemical transmitters of hormones and cytokines. This dynamic adaptation of the unity of being takes place through feedback loops between the axes, adjusting to the internal circumstances of the human body and the external conditions of the ecosystem. Thus, the unity of being is configured, establishing a direct and continuous relationship with the environment. (Source: Own elaboration).

In 1891, Smith G. P. (2000) initiated research on digestive processes in dogs in his own laboratory, work that would earn him the Nobel Prize in physiology. His findings demonstrated that psychic variables influence physiological reactions, marking the beginning of his explorations into classical conditioning (Bykov et al., 1958). Subsequently, in 1975, Ader and Cohen (González-Díaz et al., 2017) described the integration of the psyche into the NIES, considering the psyche as a key determinant of biological response. This integration gives rise to a unique system called the psychoneuroimmunoendocrine system (see Figure 3), responsible for adaptive responses to various influences, both external and internal.

Selye (1990) defines stress as a nonspecific response to a demand imposed on the body Selye H. (1946). When the compensatory mechanism fails, regardless of its origin, it exhibits fundamental characteristics of “dishomeostasis,” according to Goldstein, in the context of critical illness and chronic disease. These aspects support that dysfunction of the nervous system, known as dysautonomia, along with inflammation and dishomeostasis, are central mechanisms underlying the development and perpetuation of multiorgan failure (Toner et al., 2013; Zalewski et al., 2018).

## Pathophysiology of long COVID (post-acute sequelae of SARS-CoV-2, SPAC)

Integrating the explored concepts (synthetic physiology, neurogenic dystrophy, inflammatory reflex, neuroimmune circuit, central and peripheral sensitization, and neurogenic inflammation as part of the nonspecific response) provides new elements for understanding the pathophysiological mechanisms triggered by SARS-CoV-2 injury that persists over time with Long COVID (Post-Acute Sequelae of SARS-CoV-2, SPAC).

Chronic inflammation is a common denominator in the pathophysiology of various chronic diseases such as diabetes, cardiovascular, respiratory, mental, epilepsy, obesity, and autoimmune diseases, among others (Tracey, 2002). The neuropathology of PACS is characterized by involvement of the nervous system (NS), dysautonomia, and subsequent neurogenic inflammation (Li et al., 2023). This process is imperative in the development of PACS and contributes to the understanding of related chronic diseases.

Publications correlate Myalgic Encephalomyelitis/Chronic Fatigue Syndrome (ME/CFS) with PACS, manifesting overlapping symptoms between both pathologies (Engel et al., 2022). In the early 20th century, Speransky (Speransky, 1954) proposed that a nonspecific stimulus associated with injury, either by its intensity and/or frequency, could trigger neurogenic dystrophy with disseminated encephalomyelitis, as demonstrated in animal experimental models.

Similarly, ME/CFS can be preceded by nonspecific triggering factors, such as viral, bacterial, parasitic (acute or chronic) infections, toxic exposures, vaccination, and trauma (physical or emotional) (DynaMed, n.d.). Other functional somatic syndromes without apparent cause, such as irritable bowel syndrome, fibromyalgia, temporomandibular disorders, vulvodynia, and interstitial cystitis, have been considered as dysautonomias (Meade and Garvey, 2022). Although the relationship is not linear, dysautonomic activation and hyperinflammatory reflex are common patterns in the expression of these syndromes.

**Figure 4:**
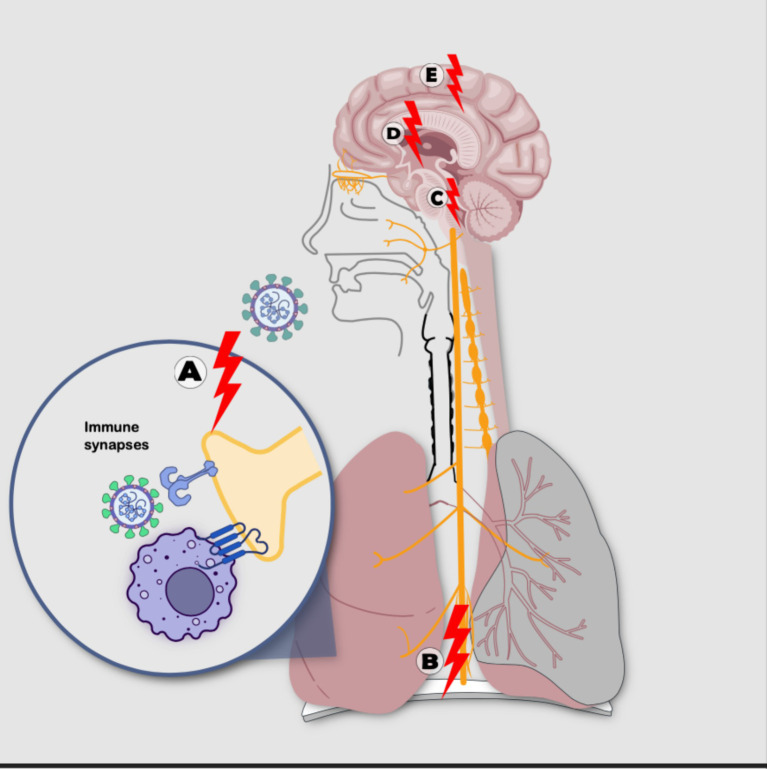
Sensitization processes: with nonlinear mechanisms following SARS-CoV-2 infection including: **(A)** sensitization-irritation of peripheral receptor through immunological synapse with immune cell or directly by the virus, **(B)** sensitization-irritation of the vagus nerve, **(C)** sensitization-irritation of the brainstem solitary tract nucleus and parabrachial regions, **(D)** sensitization-irritation of the limbic system, **(E)** sensitization-irritation of the cortex (Source: Own elaboration).

*VanElzakker* hypothesizes that during acute SARS-CoV-2 infection, the terminal axons of the vagus nerve (VN) and other nerves in the respiratory epithelium become sensitized by immune factors. The reflex response involves various levels of the NS, such as the dorsal brainstem, the Nucleus Tractus Solitarius, and parabrachial regions with glial activation, manifesting systemic symptoms like fatigue, fever, myalgia, among others. These symptoms are common in the clinical expression of ME/CFS and PACS. As part of this reflex response, a “corticalization” pathway is observed, where peripheral sensitization spreads centrally, transmitting inflammatory signals from VN terminals to the brainstem, limbic system, and neocortex (Figure 4) (VanElzakker, 2013).

The brainstem plays a fundamental role in the neural phase of the neuroimmune circuit and in the process of central sensitization, characterized by dysfunctional signaling of this component, which could be a key factor in PACS symptoms (Proal and VanElzakker, 2021). In autopsies of corpses affected by SARS-CoV-2, immune activation has been identified in the brainstem (Solomon, 2021). Furthermore, various studies have reported functional and structural abnormalities of the brainstem in ME/CFS (Shan et al., 2020), along with glial activation and cognitive impairment (Nakatomi et al., 2014). It is important to note that central sensitization of the brainstem can also be triggered by infections and inflammatory events “occurring outside the NS,” such as nonspecific injurious stimuli (Proal and VanElzakker, 2021).

Within the NS, mast cells and microglia are activated in response to SARS-CoV-2 and other viruses. The inflammatory cascade is largely maintained by exposure to “multiple hits,” which are diverse inflammatory events that collectively amplify their signaling (Proal and VanElzakker, 2021). Once activated, these immune cells retain a prepared functional state, leading to an even more robust response to subsequent challenges. This prepared state may also be crucial in symptoms such as sensory sensitivity in individuals who have survived acute neuroinflammatory events, such as encephalitis or concussion, or who may have low levels of persistent or latent neurotropic pathogenic microorganisms, including the herpes virus, among others (Proal and VanElzakker, 2021).

Any stimulus inducing the release of proinflammatory cytokines in a region of the body innervated by the VN can initiate or perpetuate this pathological response and associated chronic symptoms (Proal and VanElzakker, 2021).

## Specific responses to SARS-CoV-2 infection in the pathophysiology of PACS

The specific responses to SARS-CoV-2 infection that contribute to the development of SPAC include: direct tissue damage in one or multiple organs, the persistence of SARS-CoV-2 reservoirs in certain tissues, reactivation of neurotrophic pathogens such as herpesviruses under conditions of immune dysregulation induced by SARS-CoV-2, interactions of SARS-CoV-2 with the host’s microbiome/virome communities, and autoimmunity due to molecular mimicry between the pathogen and host proteins (Proal and VanElzakker, 2021).

### Direct tissue damage

SARS-CoV-2, similar to other virulent coronaviruses, initially enters through the respiratory epithelium using the angiotensin-converting enzyme 2 receptor. These receptors are expressed in cells of the respiratory system, brain endothelium, vascular smooth muscle, as well as gastrointestinal epithelial cells, pancreatic cells, and renal podocytes (Hamming et al., 2004). Although the mechanism of extrapulmonary spread is not yet clear, direct tissue damage from infection may be a primary mechanism contributing to long-term complications (Wiersinga et al., 2020).

Neuroinfection and subsequent neuroinflammation caused by SARS-CoV-2 have been documented in autopsy models, animal trials, and organoids. Infection can spread hematogenously through the blood–brain barrier or through the mechanism known as the “Trojan Horse,” in which immune cells infected with intracellular pathogens are actively transported to the central nervous system. Additionally, retrograde transport of the virus through neuronal axons has been observed, originating from the olfactory, glossopharyngeal, or vagus nerves (Proal and VanElzakker, 2021). In some patients with neuroinflammation and conditions like ME/CFS, the possibility of viral or bacterial pathogens infecting the vagus nerve has been established (VanElzakker, 2013).

### Persistent infection

In some cases, persistent symptoms may be related to prolonged infection with SARS-CoV-2, where the virus is not cleared for extended periods. The presence of viral reservoirs in tissues is evidenced by traces of positive PCR and amplified CD8 T-cell responses against SARS-CoV-2. Despite obtaining a negative PCR, the virus may persist in tissues, a phenomenon observed with other neurotropic viruses (Plebani, 2021; Proal and VanElzakker, 2021), thus perpetuating the low-grade chronic immune response and inflammation.

Autopsies have revealed persistent infection by SARS-CoV-2 in the human nervous system (Stein et al., 2022). Both SARS-CoV-2 and other neurotropic pathogens could be reactivated by acute SARS-CoV-2 infection. These pathogens rarely persist in the blood, typically being identified in tissues or nerves (Proal and VanElzakker, 2021).

### Molecular mimicry and autoimmunity

Infection with SARS-CoV-2 can trigger “autoantibodies” due to similarities in sequence with proteins or metabolites derived from the virus and the host’s own tissues. An important aspect in the study of PACS involves the analysis of the immune system and these autoantibodies as part of the underlying mechanisms (Proal and VanElzakker, 2021).

### Genetic predisposition and immune response

Genetic variations in the immune response, coagulation, or expression of human endogenous retroviruses are associated with an increased risk of developing PACS. However, in a study involving twins, non-heritable factors were found to determine more than half of the variability in immune parameters. These findings highlight how at least one type of microbial exposure can significantly modulate the overall immune profile of healthy individuals (Brodin et al., 2015).

### Microbiota/virome and neuroimmune dysregulation

The human body’s microbiome consists of diverse and abundant microorganisms. During SARS-CoV-2 infection and PACS, changes in the composition of the intestinal microbiome have been identified (Liu et al., 2022). This dysbiosis of the microbiota/virome can have a profound impact on the host’s genetics, immunity, metabolism, hormones, and nervous system (Ursell et al., 2012). During acute SARS-CoV-2 infection, some microorganisms undergo changes in their balance. This alteration may persist, allowing inactive pathogens to reactivate, colonize new sites, and trigger chronic symptoms (Proal and VanElzakker, 2021).

In the “multiple-hit model,” one pathogen can support the virulence of the next infection (Proal and Marshall, 2018). In the acute progression of SARS-CoV-2 or PACS, persistent pathogens can be considered predisposing factors (Proal and VanElzakker, 2021), as they overload the neuroimmune circuit, predisposing it to a state of alertness or irritation (Pavlov and Tracey, 2012; Proal and VanElzakker, 2021).

Human bacteria have been shown to play a role in the production and/or consumption of various neurotransmitters such as norepinephrine, dopamine, serotonin, and gamma-aminobutyric acid (GABA) (Galland, 2014). Furthermore, proteins and metabolites derived from the microbiota/virome influence the activity of immune cells. Alterations in host signaling or the permeability of the gastrointestinal epithelial barrier, resulting from dysbiosis, could be contributors to the onset of PACS (Proal and VanElzakker, 2021).

### Hydration, nutrition, and oxidative stress

Hydration and nutritional status are fundamental pillars of the body’s capacity to respond to various stimuli. Dehydration and nutritional deficiencies are associated with alterations in the neuroimmunoendocrine response, stress, and aging, thus impacting general health status El-Sharkawy, et al., 2015; López Plaza, et al., 2017; Birnkrant, et.al., 2018; Moszak, et al,. 2020; Zhao, et al., 2020; Ciebiera, et al., 2021; Arab, et al., 2023; Schloss JV, 2023.

The evidence supporting the importance of stress-induced deficiencies in nutrients such as magnesium, zinc, iron, calcium, and niacin is strong (Ramsey et al., 2020). While it is crucial to acknowledge the impact of individual nutrients, it’s essential to understand that the biological response to stress cannot be simplified to a single nutrient The synergistic interaction of macronutrients and micronutrients, encompassing high-quality sources of carbohydrates, fatty acids, and amino acids, along with vitamins, minerals, antioxidants, enzymes, coenzymes, and the contributions of phytochemicals, supports optimal biological function.

Maintaining adequate hydration and nutrition enhances resilience and improves the ability to adapt to both internal and external stressors. In the specific case of PACS, adequate hydration is an important factor in complete recovery (Barrea et al., 2022).

Some foods have shown notable effects on inflammatory pathways and have the potential to modulate inflammatory imbalances. Therefore, the careful selection of anti-inflammatory foods, while avoiding those with pro-inflammatory potential, is recommended as a fundamental strategy to alleviate diseases characterized by a significant inflammatory component in their pathophysiology. Moreover, a diet rich in anti-inflammatory nutrients, such as the Mediterranean diet, may prove beneficial in ameliorating sequelae secondary to COVID (Ricker and Haas, 2017; Ramsey et al., 2020).

Several clinical trials confirm the positive response of monotherapy with 22 different nutrients (cobalamin, calcium, zinc, thiamine, pyridoxine, asparagine, magnesium, niacinamide, riboflavin, oleic acid, glutamine, inositol, choline, selenium, vitamin D, iron, taurine, phosphorus, ascorbate, bioflavonoids, N-aceyl cysteine) on the probability of contracting COVID-19 and the severity of the disease (Ramsey et al., 2020). However, the response to monotherapy with these nutrients may be influenced by the nutritional exposome, biochemical individuality of each person, the concomitant deficiency of other nutrients and the special nutritional needs induced by the stress of dysfunction.

Supplementation with molecules like coenzyme Q10 and alpha-lipoic acid, targeting antioxidant cellular pathways, presents intriguing alternatives explored in treating conditions with chronic inflammation, such as PACS (Tasneem et al., 2019; Akanchise and Angelova, 2023).

A wide variety of medicinal plants, such as *Camellia Sinensis, Tripterygium Wilfordii Hook F, and Zingiber officinale*, among others, display anti-inflammatory effects (Bustamante et al., 2023). Essential oils from species such as *Eucalyptus, Cinnamomum,* and *Juniperus* exhibit therapeutic potential in modulating immunity, reducing inflammation, and exerting antiviral effects. These plants and oils contain various phytochemicals, including phenolics, terpenoids, and alkaloids, which individually exhibit anti-inflammatory, immunomodulatory, and antiviral properties with curative potential for COVID-19 (Barletta et al., 2023).

The hydration and nutritional status significantly influence disease expression. While discussing the entire spectrum of therapeutic uses of diets, nutrients, phytochemicals, and essential oils is extensive, certain key areas and their direct impact on the neuroimmunoendocrine system and associated biological responses merit attention.

## Neuraltherapeutic medicine

Neuraltherapeutic Medicine (NTM) (more commonly known as Neural Therapy) arises from the conjunction of understanding the synthetic physiology of Nervism with the discovery of the modulating effects of Local Anesthetics (LAs) on the nervous system, known as the neuraltherapeutic effect.

Researchers such as Speransky, Bykov, Orbeli, and Vischnevsky confirmed Pavlov’s experimental findings and used infiltrations of LAs, known as “novocainic blocks,” to address nervous system dysfunctions (Забродин, 1999). Rather than a simple transient anesthetic block, they described a lasting neuraltherapeutic effect that persists after the direct pharmacological effect, stimulating natural regulatory reflexes, such as anti-inflammation, that were somehow dysregulated. This approach allowed them, in a surgical context, to manage critical acute conditions such as septic or hypovolemic shock in war victims (Sechenova et al., 1993) and to modulate inflammation in both acute and chronic inflammatory and infectious pathologies, in both humans and animal models (Ariza Tarazona et al., 2020).

In Germany, a medical school named “Neural Therapy according to Huneke” was born, later enriched by scientific research from around the world. It presents itself as a therapeutic option to modulate the reflexes of the nervous system. In Colombia, a new school emerged in the 1970s, led by Julio Payán, which not only integrates Russian nervism and the foundations of the German school but also undergoes conceptual and scientific enrichment from the complexity sciences, leading to a change in its name to Neuraltherapeutic Medicine (Bustamante et al., 2023).

This school distinguishes itself by incorporating into its theoretical framework the concept of stimulating the psychoneuroimmunoendocrine system to modulate the nonspecific pathophysiological mechanism of neurogenic dystrophy and, consequently, inflammation. Additionally, it integrates reflex mechanisms of central (including the concept of corticalization) and peripheral sensitization in the process of neurogenic inflammation. These concepts manifest coherently within the semiological approach to patients and in diagnostic and therapeutic orientations, which focus depending on the level of irritation (local, segmental, or meta-segmental) (Ariza Tarazona et al., 2020).

Neuraltherapeutic Medicine, through stimuli applied to anatomical structures, usually through the use of Local Anesthetics (LAs) such as low-concentration procaine (between 0.5 and 1%), modulates the regulatory and plastic functions of the nervous system. Such application of procaine is usually very well tolerated, with minimal effects in patients such as transient dizziness and metallic taste (Dosch and Dosch, 2007). There is evidence of the neuroimmunomodulatory action of LAs, more extensively studied in inflammatory conditions and pain (Akimov and Kositsyn, 2005; Engel et al., 2022; Bustamante et al., 2023; Vinyes et al., 2023). Furthermore, other pathways of action on the information system that have not been completely elucidated have been indicated, such as influence through the microtubules of the living matrix (Cruz, 2011; Liu and Duricka, 2022; Bustamante et al., 2023).

Several authors have explored the therapeutic effect of Local Anesthetics (LA) on the Post-Acute Sequelae of COVID-19 (PACS), albeit diverging from the conceptual framework of Neuraltherapeutic Medicine. For instance, Liu reported symptom resolution in two PASC patients through the use of LA in two consecutive procedures near the stellate ganglion. Galvin conducted a retrospective review of medical records involving 195 PACS patients, noting statistically significant improvements in most symptoms following stellate ganglion block (Galvin et al., 2023). Typically, 5 mL of local anesthetic is used for this procedure (Restrepo-Garcés et al., 2012). Both authors describe the therapeutic effect as sympathetic blockade and acknowledge a lack of understanding of mechanisms beyond the anesthetic (Liu and Duricka, 2022).

Stellate ganglion block (SGB) can lead to various complications, including Horner’s syndrome and potential impacts on nearby nerve structures like the recurrent laryngeal nerve, resulting in dysphonia, dysphagia, and dyspnea. Major risks involve inadvertent vascular injection causing seizures and cardiovascular toxicity, and neuroaxial block, often requiring immediate support. Other potential complications include esophageal perforation and thyroid puncture, which may lead to neck hematomas (Restrepo-Garcés et al., 2012).

Vinyes et al. (Vinyes et al., 2022), specifically, detailed a successful Neuraltherapeutic Medicine approach in a PACS patient, involving three procedures over 8 weeks with a 16-week follow-up. While Liu and Galvin follow a linear thinking approach with a standardized, non-individualized procedure, Vinyes considers a nonlinear approach, investigating the patient’s past irritations throughout their life history, thus defining therapeutic orientations under this rationale.

The experience with neural therapeutic approaches, documented across centers in Switzerland (Mermod et al., 2008), Germany (Joos et al., 2011), Spain (Roca et al., 2010), and Colombia (Sarmiento Rodríguez, 2014), among other countries, has yielded promising outcomes in managing diverse chronic conditions. Presently, 14 scientific associations are affiliated with the International Federation of Medical Associations of Neural Therapy (IFMANT) (https://www.ifmant.at/es/), n.d. underscoring the increasing global recognition and adoption of this therapeutic modality.

## Other neuroimmune modulation strategies

Few therapeutic advances address the modulation of the neuroimmune circuit. Some studies emphasize the role of the vagus nerve (VN), a key representative of the parasympathetic nervous system, and the stimulation of the anti-inflammatory cholinergic efferent arm through implantable and external electronic devices, but these are costly and have limited access (Johnson and Wilson, 2018). Other researchers have explored the possibility of preventing neurogenic dystrophy by using adrenergic blockers during injury (Akimov and Kositsyn, 2005), but these remain limited.

Neurofeedback and biophotomodulation are potential non-invasive therapeutic tools in modulating inflammation of neurogenic origin, although there are no specific studies on their impact on the inflammatory reflex, these techniques have shown improvement in diseases with an inflammatory component and oxidative stress of the nervous system. Such as Alzheimer’s and Parkinson’s (Berman and Nichols, 2019).

## Physical exercise in inflammatory disorders

The proper prescription of exercise holds potential benefits for inflammatory disorders and is essential in managing PACS (Metsios et al., 2020; Chuang et al., 2024).

## Integral synthesis

Technological advancement has provided tools to delve deeper into the understanding of the nervous system. Although this system exhibits phenomena that are not fully explainable, the growing effort to integrate other branches of knowledge, such as quantum physics, into living systems (Tuszynski, 2020), reflects broad and ongoing scientific development.

Beyond reductionism, multiple interrelated non-linear connections are glimpsed in biological circuits following SARS-CoV-2 infection, prolonging symptoms. The properties of complex systems are not fully understood by analyzing isolated parts. Studying the organism as a whole, similar to the nervous system, represents a modernization of the biomedical paradigm.

## Conclusion

In the pathophysiology of PACS, various hypotheses are proposed that involve common patterns of nonspecific response, such as inflammation and dysautonomia. These patterns may compromise the function of the neuroimmune-endocrine system, leaving it hypervigilant, primed, or hyperexcited, triggering neurogenic inflammation following neurogenic dystrophy. Although these patterns are also described in functional syndromes, from the perspective of nervism and Pavlovian synthetic physiology, they are involved in all pathophysiological processes.

Expanding the paradigm involves recognizing the crucial role of information systems in integrating the unity of organisms. Although the existential dilemmas of the psyche or soul and their physiological repercussions are not delved into, emerging approaches in medicine include innovative concepts of the neuroimmune-endocrine system in modulating all processes of the living body. It is worth highlighting the process of inflammation and tissue repair as a cornerstone of health and disease.

Neurogenic tissue dystrophy underlies the local, segmental, or distant clinical expression. This process resembles the model of central and peripheral sensitization. The described segmental irritation refers to the classic anatomical model of deep and superficial innervation, related in a transversal reflex arc from the somite to the medullary level. On the other hand, neuromodulatory trigger points correspond to polysegmental anatomical circuits, temporal associations, and other information interactions not yet described.

Neuraltherapeutic stimulation transcends the direct pharmacological response provided by the transient anesthesia of Local Anesthetics (LA). Potentially, this stimulation modulates nervous influx in tissues, simultaneously influencing physiological and pharmacological responses, especially in inflammatory processes. This capability involves modulating the non-specific response of the Neuroimmune-Endocrine System (SNIE), enhancing the resilience of the biological system independently of the specific mechanisms of the disease cause. This therapeutic modality could be potentially useful in pathologies with neurogenic inflammation, such as Post-COVID-19 Accumulative Syndrome (PACS).

More details about the Neural therapy should be given/discussed: the putative side effects of the blockade of the stellate ganglion, whether or not these procedures are routinely done in known Medical centers.

Examining points of convergence between the neuroscience paradigm and Neuraltherapeutic Medicine (NTM) allows for a more integrative synthesis, providing a fuller perspective on information systems and their interaction. Although much is yet to be understood, especially regarding the psyche, clinical practice offers a fertile ground for exploration and advancement in this field.

## Gratitude

To the living God, whose wisdom enlightens our understanding of His majestic creation, for the service of humanity. To the Church of God Ministerial of Jesus Christ International, a guiding light on my path, serving as my guide to communion with the almighty on the journey toward eternity.

## Author contributions

CB: Writing – review & editing, Writing – original draft, Conceptualization. LBPB: Writing – review & editing. JCR: Writing – review & editing.
